# Co-drawing of technical and high-performance thermoplastics with glasses via the molten core method

**DOI:** 10.1038/s41598-023-32174-7

**Published:** 2023-03-29

**Authors:** Clément Strutynski, Raphaël Voivenel, Marianne Evrard, Frédéric Désévédavy, Gregory Gadret, Jean-Charles Jules, Claire-Hélène Brachais, Frédéric Smektala

**Affiliations:** Laboratoire Interdisciplinaire Carnot de Bourgogne, UMR 6303, CNRS-Université de Bourgogne-Franche-Comté, 9 Avenue Alain Savary, 21078 Dijon, France

**Keywords:** Materials science, Materials for devices, Materials for optics, Soft materials

## Abstract

Among the different fundamental aspects that govern the design and development of elongated multimaterial structures via the preform-to-fiber technique, material association methodologies hold a crucial role. They greatly impact the number, complexity and possible combinations of functions that can be integrated within single fibers, thus defining their applicability. In this work, a co-drawing strategy to produce monofilament microfibers from unique glass-polymer associations is investigated. In particular, the molten core-method (MCM) is applied to several amorphous and semi-crystalline thermoplastics for their integration within larger glass architectures. General conditions in which the MCM can be employed are established. It is demonstrated that the classical glass transition temperature compatibility requirements for glass-polymer associations can be overcome, and that other glass compositions than chalcogenides can be thermally stretched with thermoplastics, here oxide glasses are considered. Composite fibers with various geometries and compositional profiles are then presented to illustrate the versatility of the proposed methodology. Finally, investigations are focused on fibers produced from the association of poly ether ether ketone (PEEK) with tellurite and phosphate glasses. It is demonstrated that upon appropriate elongation conditions, the crystallization kinetics of PEEK can be controlled during the thermal stretching and crystallinities of the polymer as low as 9 mass. % are reached in the final fiber. It is believed such novel material associations as well as the ability to tailor material properties within fibers could inspire the development of a new class of hybrid elongated objects with unprecedented functionalities.

## Introduction

Multimaterial fibers are considerably boosting the development of new generations of miniaturized devices and components with applications spanning all technological domains of interest (health, energy, electronics, environment, defense, telecommunications, etc.). The fabrication of such remarkable and undeniably useful objects relies on advanced material shaping processes, which are necessary to simultaneously embed multiple functionalities within fibers. Owing to strong contributions from the material science community, the range of materials that can be integrated into fiber assemblies^1^, as well as their possible combinations and the way they are arranged within the elongated structures have now immensely widened^2–5^. To achieve this, numerous preform preparation strategies and unconventional thermal drawing techniques were implemented, such as the stack-and-draw technique^6,7^, insertion methods^8,9^ (derived from the rod-in-tube^10,11^), extrusion^12^, additive manufacturing^13–15^ as well as the direct wire insertion^16,17^, and so on. Among those popular multimaterial fiber preparation strategies, the molten core method (MCM) appears as one of the main attractive solutions for the design of intricate hybrid elongated structures^18^. The MCM relies on the following principle: a core material is inserted within an amorphous cladding to form a macroscopic preform and the assembly is subsequently stretched into thin continuous fibers using conventional fiber-drawing equipment, with the particularity that during the stretching procedure, the core material is in a molten state, while the amorphous cladding material is only softened. In other words, the cladding acts as a support to restrict the flow of the liquid core material. In the early development of the molten core technique^19^, the method referred to thermal elongation procedures during which chemical interactions within the core materials mix or between the molten core and the cladding would occur. This process would allow the production novel materials that could not be synthesized otherwise or could not be integrated into fibers through the conventional drawing process. The technique now designates, in a broader way, thermal elongation procedures during which a core material is drawn while being in the molten state^20^. To this date, the MCM has mainly been exploited for the in-fiber incorporation of inorganic, non-conventionally stretchable materials (glass compositions with high rare-earth concentrations, semi-conductors, metals, etc.), but the integration of polymers into glass-based elongated structures using the same methodology has not been studied.

It is proposed in this work to assess for the first time the fiber drawing ability of selected thermoplastics using the molten core method. First, the conditions in which the MCM can be applied to integrate polymeric materials within larger glass frames are discussed, and general thermal parameters are established. Following, experimental investigations are carried out and several fibered structures produced from unprecedented glass/polymer associations are examined. It is demonstrated that amorphous and semi-crystalline polymers can be co-drawn with low glass transition temperature oxide glasses while being in a low viscosity regime. This result constitutes an important breakthrough as glass/polymer associations are almost exclusively limited to the combination of chalcogenide glasses and high-performance amorphous thermoplastics. In a final section, investigations concerning the production of continuous poly ether ether ketone (PEEK) fibers are presented. Due to the wide MCM temperature range of PEEK, it is successfully elongated in combination with several oxide glass claddings of different compositions (tellurites and phosphates). The quenching effect caused by the thermal drawing was also investigated towards the control of the degree of crystallinity of PEEK fibers.

## Experimental section

### Glass synthesis and preform preparation

All glasses considered in this work were fabricated using the standard melt-quenching procedure: TeO_2_ (Fox chemicals 99.9%), ZnO (Alfa Aesar 99.99%) Na_2_O (Alfa Aesar 99.99%), La_2_O_3_ (Alfa Aesar 99.99%) and Y_2_O_3_ (Alfa Aesar 99.99%) in the powder form were weighed in adequate ratios and put together inside a platinum crucible. The precursor mix was then heated up to 800–900 °C depending on the composition of the glass, and the temperature was maintained at this value for one hour. Following, the hot melt was poured into a preheated brass mold of chosen architecture so that glass parts with adequate shapes were produced (cylindrical, parallelepipedal, etc.). The glass was then annealed at *T*_*g*_—10 °C for 5 h and its temperature was slowly (≈2 °C/min) ramped down to room temperature. The preforms were then fabricated using a combination of various methods (molding, mechanical drilling, stacking, insertion, etc.). Glass pieces are assembled together with various polymer parts (rods and films), which were bought from different suppliers: PEEK and PEK rods (Ensinger), PES films (GoodFellow), PLA, PETG, ABS, PC and PA 6–6 filaments (Ultimaker), see Table 1 for the meaning of the used abbreviations.

**Table 1:** Glass transition temperature *T*_*g*_, onset crystallization temperature *T*_*c*_, melting temperature *T*_*m*_ and decomposition temperature *T*_*d*_ of various amorphous and semi-crystalline thermoplastic polymers.

Polymer	Name	Nature	T_g_ [± 3 °C]	T_c_ [± 3 °C]	T_m_ [± 3 °C]	T_d_ [± 3 °C]
Polylactic acid	PLA	Semi-crystalline	59	87	150	309
Acrylonitrile butadiene styrene	ABS	Amorphous	105	–	–	334
Polyethylene terephthalate glycol	PETG	Amorphous	79	–	–	373
Poly amide (Nylon)	PA6-6	Semi-crystalline	62	-	185	363
Polycarbonate	PC	Amorphous	115	-	-	410
Poly ether sulfone	PES	Amorphous	223	-	-	549
Poly ether ketone	PEK	Semi-crystalline	168	-	375	553
Poly ether ether ketone	PEEK	Semi-crystalline	167	174	337	616

### Differential scanning calorimetry (DSC)

DSC experiments were performed on a TA Instruments Q1000 system with standard aluminum pans. Temperature and energy calibrations of the system were realized using Indium (*T*_*m*_ = 156.6 °C) and sapphire. For all the PEEK samples (5–10 mg), the analysis method starts with a 5 min isothermal step at 23 °C followed by a heating ramp at 10 °C/min to 450 °C and a last 5 min isothermal step. The baseline was recorded in the same conditions and subtracted from each thermogram before analysis, using Thermal Analysis™ software (www.tainstruments.com). DSC experiments during the first heating lead to the determination of the glass transition temperature *T*_*g*_, the melting temperature *T*_*m*_, the melting enthalpy ΔH_m_, the cold crystallization temperature T_cc_, the cold crystallization enthalpy ΔH_cc_, and the degree of crystallinity χ_c_. The degree of crystallinity, χ_c_, is calculated from Eq. (1) for samples showing a cold crystallization temperature. The value of the melting enthalpy for a 100% crystalline PEEK ΔH_m_^100%^ considered is 130 J/g and taken in Blundell et al. work^21^.

Equation (1):

$$\chi c= \frac{\Delta Hm-\Delta Hcc}{{\Delta H}_{m}^{100\%}}$$

The values of ΔH_m_, ΔH_cc_ and χ_c_ given in Table 4 are averaged from three different DSC measurements. The average standard deviation for χ_c_ is determined from those multiple measurements and is found to be 2%.

### Thermogravimetric Analysis (TGA)

TGA experiments were performed on a TA Instruments Q600 system with standard platinum pans. The measurements were carried out by heating ≈15–30 mg samples from room temperature up to 700 °C at a 10 °C/min rate and under a 50 mL/min nitrogen gas flow. The decomposition temperature *T*_*d*_ of the considered polymer corresponded to the temperature at which a 2% mass loss was reached on the TGA curve.

### Dynamic mechanical analysis (DMA)

Viscosity curve of polymer samples was determined through unidirectional cylindrical compression model via a DMA system working in linear deformation mode^22^. DMA measurements were performed on few-millimeter-thick and 10 mm diameter cylindrical samples using a TA Instruments Q800 system in parallel plate configuration. The thickness of the sample was then monitored when heated at a 2 °C/min rate from *T*_*g*_—20 °C up to an upper-temperature limit. This upper limit depends on the considered material and was the temperature at which full deformation of the sample occurred. The viscosity η is calculated from Eq. (2)^22^:

$$\eta =\frac{2\pi F{l(t)}^{5}}{3V(2\pi {l(t)}^{3}+V)(1+\alpha \Delta T(t))\frac{dl}{dt}}$$

where *l(t)* (in m) was the sample thickness at a given time *t*, V (in m^3^) the sample volume, α (in K^-1^) the coefficient of thermal expansion, *ΔT(t)* (in K) the difference between room and the measurement temperature at a given time *t* and *dl/dt* (in m/s) the axial deformation rate. Note that the experiments were carried out with a constant loading force F = 2.0 N.

## Results and discussion

### Material considerations

In this first section, we establish the general conditions in which the MCM can be applied for the integration of continuous thermoplastic material inclusions within larger glass structures. From a general standpoint, thermoplastic polymers can be defined as organic materials that soften upon heating and which are classified as either amorphous or semi-crystalline. Amorphous polymers have long been used for the development of optical fibers owing to their excellent thermo-viscous properties which are compatible with the thermal drawing process. They exhibit a slow viscosity change when heated above their glass transition temperature (*T*_*g*_), which allows for flow control and thus the fabrication of milli-, micro-, or even nano-structured elongated arrangements^23,24^.

**Figure 1:**
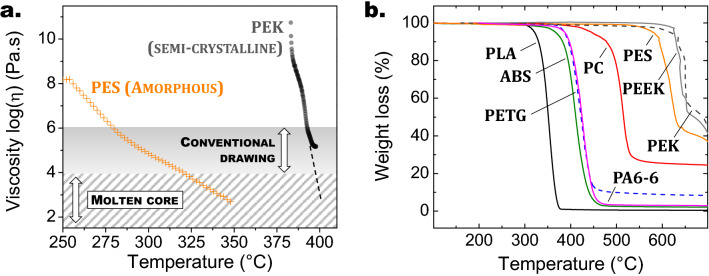
(**a**) Viscosity curves of amorphous polyether sulfone (PES) (orange symbols) and semi-crystalline polyether ketone (PEK) (black symbols). Viscosity ranges for the conventional fiber-drawing method (10^6^ to 10^4^ Pa s) and the molten core technique (η < 10^4^ Pa.s) are displayed on the graph. (**b**) TGA curves of various amorphous or semi-crystalline thermoplastic polymers (see Table 1 for the meaning of the used abbreviations).

Semi-crystalline materials on the other hand, do not soften when heated above *T*_*g*_ due to their structure being a mix of rigid crystalline phases and amorphous domains, and are therefore not suitable for fiber-drawing. PEEK is for instance known for having crystalline phases with lamellar (10 nm) or spherulitic morphologies (1-10 µm) dispersed within an amorphous matrix^25^. Upon heating, such semi-crystalline polymers rapidly transform into a low-viscosity liquid when reaching temperatures above their melting point *T*_*m*_. The rheological behaviors of the two types of thermoplastic polymers mentioned here are illustrated on Fig. 1a, where viscosity curves of amorphous PES (orange symbols) and semi-crystalline PEK (black symbols) are plotted as function of temperature. Note that PEK has a considerably sharper viscosity variation as compared to PES, with η_PEK_ decreasing from 10^8^ to 10^5^ Pa.s over a ≈10 °C temperature range while this viscosity variation occurs over a ≈100 °C temperature range for η_PES_. That kind of rheological behavior prevents semi-crystalline polymers from being stretched into fibers through the conventional drawing method.

**Figure 2:**
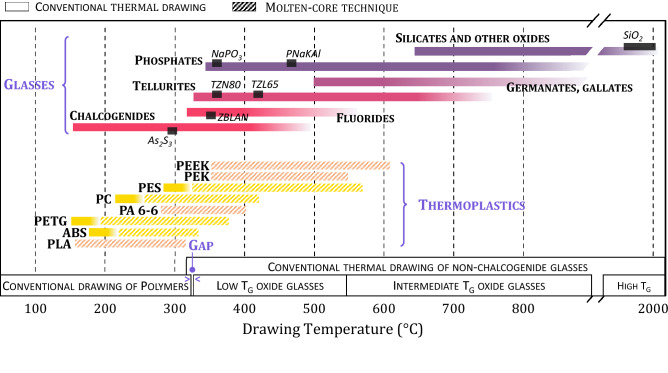
Drawing temperature ranges of various amorphous and semi-crystalline thermoplastics and glass materials using the conventional fiber-drawing method (open rectangle) or the molten core (shaded rectangle) method (see Table 1 for polymer acronyms). Drawing temperature ranges of known glass compositions (SiO_2_, As_2_S_3_, NaPO_3_ and ZBLAN) and glasses investigated in the present work (TZN80, TZL65 and PNaKAl, see Table 3 for compositions) are shown on this figure (filled rectangle). Conventional drawing temperature domains of different classes of materials (amorphous polymers, low, intermediate and high *T*_*g*_ glasses) are also shown on the bottom part of the graph. It is interesting to note that the drawing temperature domains of amorphous polymers and low *T*_*g*_ oxide glasses do not overlap.

The molten core technique can be applied to polymers when their viscosity reaches values below 10^4^ Pa.s, which is considered as the lower viscosity limit for regular thermal drawing^26^. For semi-crystalline organic materials, this corresponds to temperatures above *T*_*m*_. Based on the viscosity curve of PES shown on Fig. 1a, the MCM lower temperature limit is 320 °C, which corresponds to approximately *T*_*g*_ + 100 °C. This empirical value will be referred to in the remainder of the manuscript as a first approximation of the lower MCM temperature limit for amorphous polymers. On the other hand, the upper stretching temperature limit of both amorphous and semi-crystalline thermoplastics in the molten state can be defined as the decomposition temperature *T*_*d*_, i.e. the temperature at which a 2% mass loss is measured through thermogravimetric analysis (TGA) at a 10 °C/min heating rate. TGA curves of various thermoplastics are plotted on Fig. 1b. The corresponding decomposition temperatures are recapped in Table 1 along with other characteristic temperatures. In summary, the temperature range in which the MCM can be applied to thermoplastics is specified here as* T*_*m*_—*T*_*d*_ and *T*_*g*_ + 100 °C—*T*_*d*_ for semi-crystalline and amorphous polymers respectively. This temperature range is reported on Fig. 2 for various polymers and compared to the thermal-drawing temperature domains of several glass families.

From Fig. 2, it is clear that the MCM applied to thermoplastics not only expands the range of polymers that can be integrated into glass fibers (extension to semi-crystalline polymers), but also the range of possible glass-polymer combinations. With few exceptions^27^, glass-polymer associations have long remained limited to the embedding of chalcogenide glasses within high-performance amorphous thermoplastic claddings such as PES or PEI^23,28^. Semi-crystalline polymers on the other hand, have mainly been stretched into fibers in association with amorphous thermoplastics^29,30^ but never, to the best of our knowledge, with glasses. The methodology described here opens new opportunities for the design of intricate multimaterial fibers based on unusual materials combinations. It indeed bridges the gap that exists between the conventional drawing temperature ranges of amorphous polymers and low *T*_*g*_ oxide glasses (see Fig. 2). A panel of several glass-polymer associations for fiber fabrication is presented in the following sections to illustrate the potential of the presently described method.

### First results

**Figure 3:**
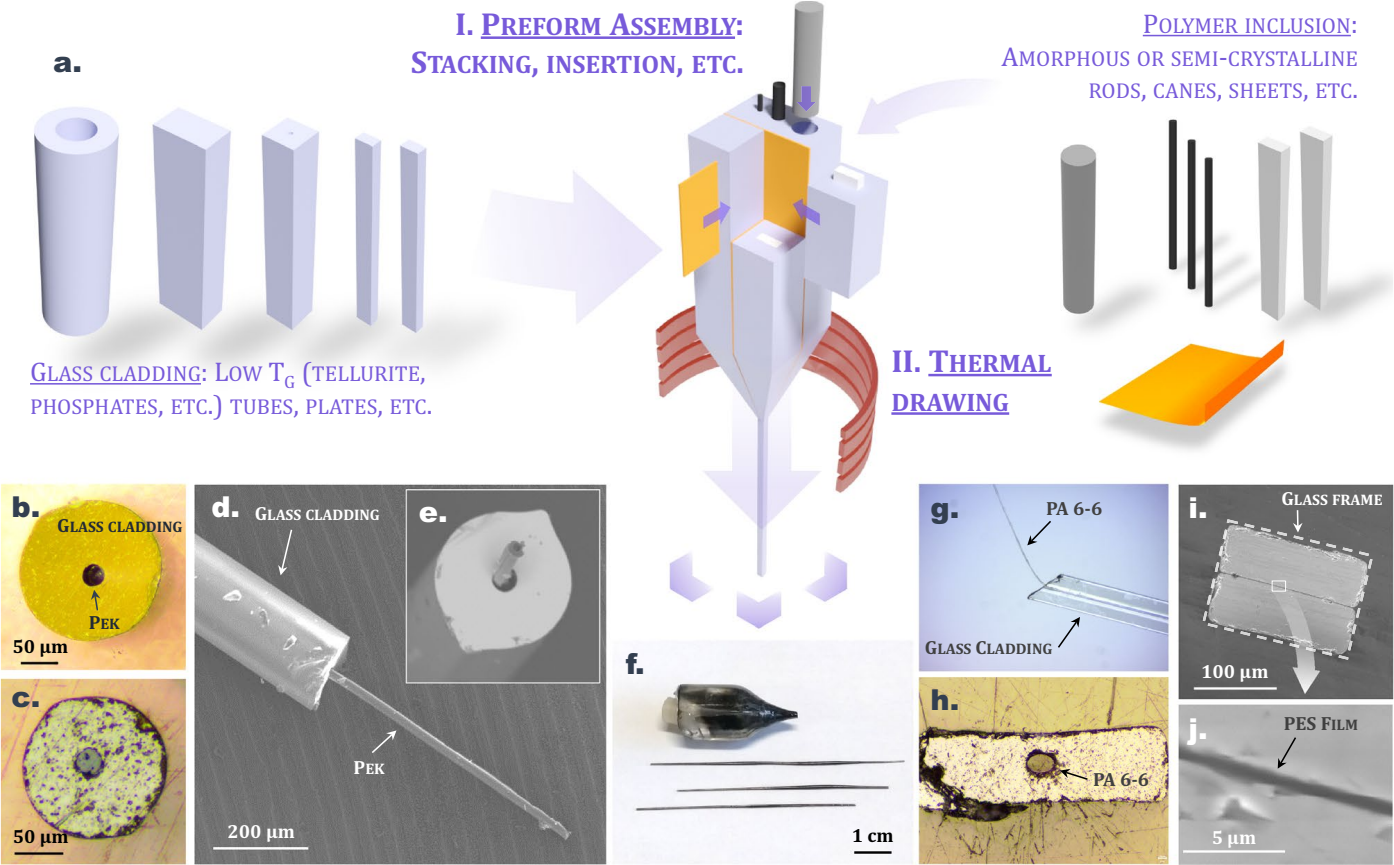
(**a**) Description of the molten core method applied to glass-polymer associations (image created using blender software version 2.92.0, www.blender.org/). Cross-sectional view of (**b**) a TZL65 glass cladding / PEK core fiber and a (**c**) TZL65 glass cladding / PEEK core fiber. (**d**) and (**e**) SEM images of a TZL65 glass cladding / PEK core fiber with the thin PEK core poking out of the glass cladding. (**f**) Picture of the neckdown region and canes from the failed stretching of a TZN60/PLA composite preform. (**g**) Longitudinal and (**h**) cross-sectional view of a rectangular TZN60 glass fiber with a central continuous Nylon inclusion. (**i**) and (**j**) SEM images (× 300 and × 10,000 respectively) of a TZN75 glass fiber integrating a thin PES structure.

In this section, several materials combinations are explored to produce elongated glass/polymer structures. The preform fabrication methodology relies on conventional preform fabrication strategies, namely the insertion technique (insertion in a tube manufactured through mechanical drilling or molding procedures), stack-and-draw, etc. Description of the general preform fabrication process and subsequent thermal drawing is described in Fig. 3a: simple glass pieces (plates, tubes, etc.) are combined with polymer rods, canes (cylindric, rectangular, etc.) and films into a macroscopic preform with arbitrary geometry which is then elongated into tens-of-meters of continuous fiber. Figure 3b–j illustrates the various glass/polymer associations that are investigated in this work. First, simple and small-size cylindrical inclusions of thermoplastic are integrated within larger glass structures. Information about the different glass/polymer combinations that are tested is summarized in Table 2.

**Table 2:** Glass transition temperature *T*_*g*_, thermal drawing temperature range *T*_*draw*_ and fabrication details of the various glass/polymer associations that are investigated in this work for the production of composite fibers.

Glass	*T*_g_ (°C)	*T*_*draw*_(°C)	Polymer—shape	Preform preparation	Result
TZN60	260 °C	335–345	PLA—8 mm rod	Molding–Insertion	Failure
TZN60	260 °C	335–345	Nylon—1 mm cane	Drilling–Insertion	Success
TZN75	308 °C	385–395	PES—50 µm film	Stack-and-draw	Success
TZL65	374 °C	410–420	PEK—1 mm cane	Drilling–Insertion	Success
TZL65	374 °C	410–420	PEEK—1.1 mm cane	Drilling–Insertion	Success

Figure 3b,c show a TZL65 glass fiber (glass compositions 65 TeO_2_–30 ZnO–5 La_2_O_3_ mol %) with PEK and PEEK cores respectively. Remarkably, the continuous organic inclusion runs all along the fiber length, as illustrated on Fig. 3d,e, where a thin continuous PEK filament is seen pocking out of a larger glass cladding. A similar successful integration of a Nylon wire within a rectangular TZN60 glass cladding (glass compositions 60 TeO_2_–20 ZnO–20 Na_2_O mol %) is depicted on Fig. 3g,h. The co-drawing of the selected materials is possible because, as discussed in the previous section, the drawing temperature of the cladding glass lies between the melting and decomposition temperatures of the considered semi-crystalline polymers. When this criterion is not respected, the organic structure risks severe degradation, as seen in Fig. 3f, which describes the neckdown region and canes resulting from the thermal stretching of a PLA rod (8 mm in diameter) within a TZN60 glass tube. The drawing temperature range of the vitreous cladding material exceeds the decomposition temperature of PLA (350–370 °C against 309 °C respectively), which leads to the deterioration of the thermoplastic that turns out completely carbonized after the drawing process. Indeed, those two materials do not appear compatible for the MCM from the diagram given in Fig. 2.

Finally, a more complex sandwich-like fiber architecture is produced from the combination of Polyether sulfone and a TZN75 tellurite glass (glass composition 75 TeO_2_–20 ZnO–5 Na_2_O mol %). The preform is prepared from two rectangular glass plates and a 50 µm thick PES film using a modified stack-and-draw method^31^. SEM images of the fabricated fiber are shown in Fig. 3i,j. The thin PES structure is preserved after thermal stretching, and more remarkably its thickness is submicronic (≈0.7 µm, see Fig. 3j) and stays well uniform on the whole fiber section. No breakup due to capillary instability is observed on the fabricated elongated structures, despite the polymer ribbon meeting the requirements for it to potentially occur (namely low viscosity and submicronic thickness)^32^. Note that the two materials combined in this architecture are both amorphous but have a significant glass transition temperature difference of ≈83 °C (*T*_*g*_ = 225 °C and *T*_*g*_ = 308 °C for PES and TZN75 glass respectively), which means they possess very distinct conventional drawing temperature ranges (285–340 °C for PES and 390–410 °C for the TZN75 glass). As the TZN75 parts represent ≈99.5% of the volume of the composite preform considered here, its thermal stretching is carried out at the drawing temperature of the glass, which implies that PES is in the molten core viscosity domain during the procedure (i.e. η < 10^4^ Pa.s, see Fig. 1a). It shows that the classical thermal limitation related to *T*_*g*_ compatibility can be overcome when considering glass/polymer associations for fiber fabrication.

We have proposed highly unconventional glass/polymer associations to produce elongated architectures. Given the nature of the combined materials, fibers composed of spatial domains with important chemical reactivity contrasts (towards specific acids or solvents) could be manufactured. This aspect may be useful for further functionalization of the fabricated objects through targeted post-drawing processing procedures (redox reactions, dissolutions, selective etching, spatial segregation, etc.). The authors have for instance demonstrated that such composite assemblies enable the fabrication, through selective etching of the glass cladding, of quasi-exposed core fibers which have potential for the evanescent wave probing of liquids^33^. Also, the integration of thermoplastics could help bring new functionalities (mechanical, electrical, optical, etc.) within glass fiber structures especially if specialty composite polymers (with various loads: metals, ceramics, glass fibers, nanoparticles, conductive loads, fluorescent dyes, etc.) are considered. A wide variety of elongated objects with unprecedented functionalities could in fact be produced from the methodology proposed in this work.

### Glass cladding / PEEK core fibers

#### Fiber fabrication

**Figure 4:**
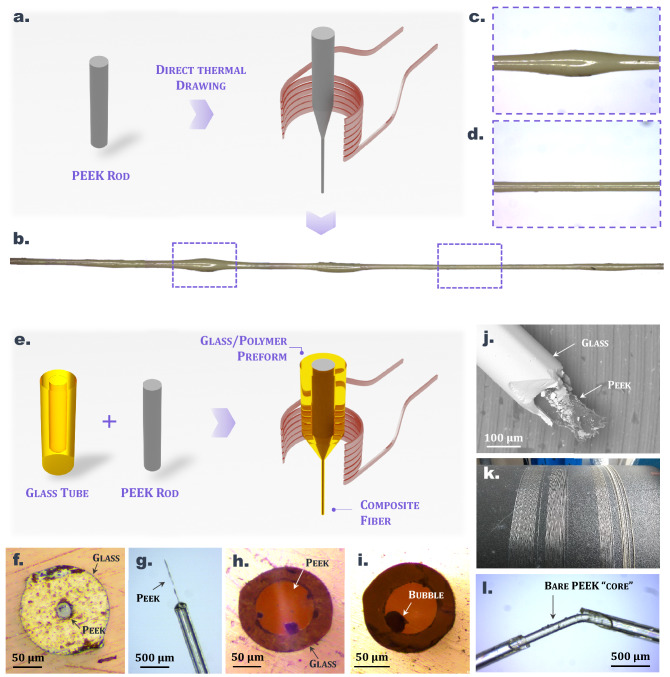
(**a**) Description of the fabrication process of a single-material PEEK fiber through the conventional drawing process (image created using blender software version 2.92.0, www.blender.org/). (**b**) Picture of a ≈5 cm long single-material PEEK fiber with a non-constant diameter. Portions of fiber with (**c**) or without (**d**) diameter irregularities (beadings). (**e**) Description of the fabrication process of a composite Tellurite/PEEK fiber through the molten core method (image created using blender software version 2.92.0, www.blender.org/). (**f**) and (**h**) cross-sectional view of the Tellurite/PEEK composite fibers with various core/cladding ratios. (**i**) Fiber with a bubble defect. (**g**) Longitudinal view of the composite fiber with the central PEEK core poking out at the fiber tip. (**j**) SEM image (× 205) of a roughly cut Tellurite/PEEK fiber. (**k**) ≈60 m of tellurite/PEEK fiber stored on a spool. (**l**) Picture of a fractured segment of the composite fiber.

In this section, we focus on the incorporation of PEEK within larger elongated glass structures using the molten core method. PEEK is a very popular material among thermoplastics, mainly because it can sustain relatively elevated temperatures, has excellent mechanical and chemical robustness as well as good biocompatibility^34^. Fiber technology could benefit greatly from the unique properties of such thermoplastics. PEEK coatings for optical fiber reinforcement are already commercially available and enable reliable operation in harsh environments. Typically, PEEK coatings appear useful for applications in aerospace or energy industries. However, due to its rheological properties, PEEK cannot be stretched into fibers using the conventional drawing technique, as described in Fig. 4a–d. In order to be shaped, this semi-crystalline polymer has to be heated above its melting temperature. Beyond this temperature, the viscosity abruptly falls (see Fig. 1a which illustrates the evolution of the viscosity of semi-crystalline PEK) and the material flow cannot be controlled. This point considerably hampers the production of fibers with uniform and preserved cross-sectional ratio. Figure 4b shows a longitudinal view of a PEEK fiber fabricated using the conventional thermal drawing procedure and which has a non-constant diameter. The formation of beadings along the fiber can be observed (see Fig. 4c) due to flow discontinuity of the polymer. In order to control PEEK flow during thermal drawing, it must be embedded within an amorphous cladding (here glass) as in the molten core method. PEEK is selected because it has a wide MCM temperature range (from *T*_*m-PEEK*_ ≈340 °C to *T*_*d-PEEK*_ = 616 °C) and can be combined with a large variety of glasses (chalcogenides, fluorides, tellurites, phosphates, etc.), as long as their drawing temperate is within the MCM temperature range of PEEK (see Fig. 2).

Here, we selected a tellurite glass cladding to apply the MCM to PEEK. First, a glass tube closed at the bottom, with 16 mm and 10 mm of outer and inner diameter respectively is fabricated using a simple casting method. The length of the fabricated glass tube is approximately 60 mm. Then, a 10 mm diameter and 60 mm long peek rod is inserted inside the tube and the assembly is subsequently thermally drawn into meters of composite fibers. A description of the process is depicted in Fig. 4e. Various views of the resulting fibers are shown on Fig. 4h–j. Structures with diverse glass-to-PEEK ratios can be obtained by changing the dimensions of the glass tube and the PEEK rod (e.g. using a glass tube with respectively 9 mm and 1.1 mm external and internal diameters, and 1.1 mm PEEK cane), as seen in Fig. 4f,g. Remarkably, the structure is well uniform, as highlighted by the length of fiber produced and depicted on Fig. 4k. The PEEK inclusion also appears continuous (see Fig. 4l) over the whole fiber length. The glass cladding, if drawn at the proper viscosity, acts as a support that restricts the flow of polymer and helps control the uniformity of the organic structure and the overall fiber cross-section.

#### Degree of crystallinity of PEEK

We now investigate the influence of the molten core method on the degree of crystallinity of PEEK when elongated within a vitreous material. During the thermal drawing, the polymer is molten and forms a liquid bath within the glass cladding just above the neckdown region. As the stretching is carried out, the molten PEEK flows through the neckdown region with the glass after which it rapidly solidifies to form the composite fiber. Because of the micrometric dimensions of the final fibered objects, both the polymer and the glass are very quickly cooled down, in a matter of few seconds, from the drawing temperature to room temperature. This treatment is equivalent to a quenching process. It is widely admitted that the crystallization kinetics of a melted polymer during cooling is directly dependent on the rate of temperature change^35,36^. For this reason, it is decisive to investigate the degree of crystallinity of PEEK in fibers fabricated using the molten core method, especially when several Physico-chemical properties are directly correlated to this parameter (density, tensile strength, heat resistance, etc.).

**Figure 5:**
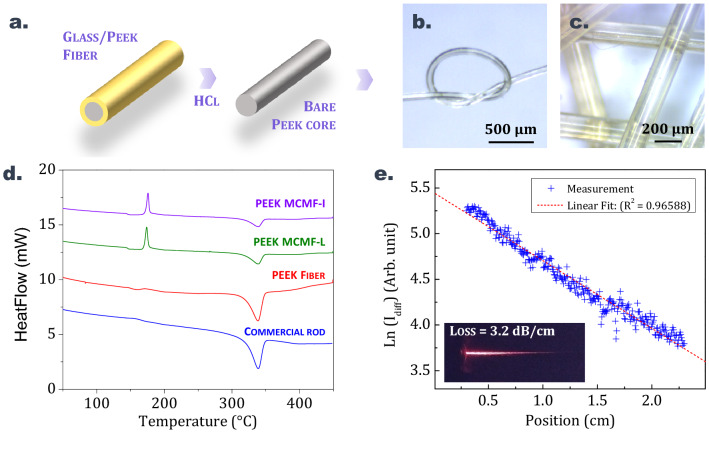
(**a**) Description of how the bare PEEK core samples are obtained from the composite fiber: fiber segments are immerged a few minutes in concentrated hydrochloric acid (HCl ≈6 mol L^-1^) in order to remove the glass cladding. (**b**) and (**c**) Pictures of uncladded and transparent PEEK fibers fabricated by the molten core method. (**d**) DSC curves from which the crystallinities of the different PEEK samples are calculated. (**e**) Optical losses measurement of a bare PEEK core at 633 nm. inset: longitudinal view of the diffused light when propagating through the PEEK waveguide.

A PEEK rod is co-drawn with three different tellurite or phosphate glass claddings which have either a low, intermediate or high glass transition temperature. Compositions of the glasses used for the MCM are recapped in Table 3 along with their glass transition temperatures and thermal drawing temperature ranges. Fibers with a comparable PEEK/glass diameter ratio of ≈0.6 are produced for this experiment with all three claddings using the methodology described in the previous section. MCM drawings are performed in similar conditions (preform feed of 0.5 mm/min, draw speed ranging from ≈1 to 5 m/min, depending on the fiber diameter). Table 4 presents the thermal transitions (*T*_*g*_, *T*_*c*_, *T*_*m*_), enthalpy of cold crystallization (ΔH_cc_), enthalpy of melting (ΔH_m_) and degree of crystallinity (χ_c_) for the starting commercial rod of PEEK, for a PEEK fiber produced using the conventional thermal drawing process (without glass cladding) and for PEEK core fibers produced using the MCM with either a low, intermediate or high *T*_*g*_ glass (noted MCMF-L, MCMF-I, and MCMF-H respectively). The degree of crystallinity is averaged from the results of three measurements for all samples (see “Experimental” section). Results for PEEK fibers of different diameters are presented in Table 4. The values are extracted from the DSC curves such as the ones plotted in Fig. 5d. It is worth mentioning that for PEEK samples coming from the MCM, the glass cladding is removed prior to the DSC measurement (as described in Fig. 5a). First, the fiber is immersed in concentrated hydrochloric acid (6 mol L^−1^) for a few minutes to dissolve the glass. Then, the PEEK core is retrieved, thoroughly rinsed in deionized water and dried at 80 °C for 1 h in a vacuum oven. Figure 5b,c show pictures of bare PEEK samples after the glass cladding has been removed. The starting material, i.e. the PEEK commercial rod as well as the fibers obtained when the polymer is elongated alone, show high degrees of crystallinity since χ_c_ values reach 30%. Similar values are measured for samples with larger diameters, i.e. for portions where beadings form along conventionally drawn PEEK fibers (noted *PEEK fiber—beadings* in Table 4). For these samples, no cold crystallization is observed during the first heating (see DSC curves in Fig. 5d), which means that the transformation process (rod fabrication and drawing) applied to the PEEK induces a sufficiently low cooling rate to allow for the crystallization of the samples to take place. Moreover, the drawing process itself induces a regular alignment of the polymeric chains in the direction of the flow^37^ leading to a highly packed and thus crystallized material. However, when the molten core method is applied to PEEK/glass systems, a strong decrease of the degree of crystallinity of PEEK is observed, with χ_c_ values decreasing down to 9 mass. % for some samples. As the solid-state organization of this polymer is known to be very sensitive to the cooling rate^35^, a high cooling rate applied to the molten PEEK results in a near-quenched material showing a low degree of crystallinity.

**Table 3:** Composition, glass transition temperature *T*_*g*_ and thermal drawing temperature range *T*_*draw*_ of the different glasses used as claddings for the MCM applied to PEEK.

MCM sample name	Glass cladding	Glass composition (mol. %)	*T*_*g*_ (°C)	*T*_*draw*_ (°C)
MCM-L	TZN80	80 TeO_2_–10 ZnO–10 Na_2_O	285	355–375
MCM-I	TZL65	65 TeO_2_–30 ZnO–5 La_2_O_3_	374	410–420
MCM-H	PNaKAl	45 P_2_O_5_–20 Na_2_O–20 K_2_O–15 Al_2_O_3_	416	465–480

**Table 4:** Diameter of the PEEK structure (Ø_PEEK_) in the considered MCM sample, cooling rates during the drawing, thermal transitions (T_g_, T_c_, T_m_), enthalpy of cold crystallization (ΔH_cc_), enthalpy of melting (ΔH_m_) and degree of crystallinity (χ_c_) of bulk and fibered PEEK samples.

Samples	Ø_PEEK_ (µm)	T_c_ (°C)	ΔH_m_ (J/g)	T_m_ (°C)	ΔH_cc_ (J/g)	χ_c_ [± 2%]
Commercial Rod	–	–	–	339	42.6	33
*Conventional drawing*						
PEEK fiber	-	–	–	339	38.4	30
PEEK fiber—beadings	-	–	–	339	38.9	30
*Molten core method*						
MCMF-L (*T*_*g*_ = 285 °C)	105	173	34.9	339	23.5	9
	145	175	41.6	339	24.9	13
	230	169	37.9	339	13.8	19
MCMF-I (*T*_*g*_ = 374 °C)	125	176	23.6	339	34.9	9
MCMF-H (*T*_*g*_ = 416 °C)	175	175	23.4	340	34.6	9

In first approximation, we consider that the cooling rate of PEEK during the molten core method solely depends on the drawing temperature of the cladding glass and the draw speed. For a fixed drawing speed, i.e. for a given PEEK core diameter, samples produced using a higher *T*_*g*_ glass cladding yield lower degrees of crystallinity. As described in Table 4, when considering fibers with similar diameters, samples drawn from an intermediate *T*_*g*_ glass cladding (MCM-I, diameter 125 µm) exhibit lower degrees of crystallinity when compared to samples drawn from a low *T*_*g*_ glass cladding (MCM-L, diameter 145 µm). In that case χ_c_ values are respectively 9 and 13%. On the other hand, for a fixed temperature of elongation, i.e. for a given MCM glass cladding, fibers with smaller diameter yield lower degrees of crystallinity due to higher drawing speed. MCM-L samples with PEEK cores of 105, 145 and 230 µm indeed show a degree of crystallinity of respectively 9%, 13% and 19% (see Table 4). To some extent, those results demonstrate that the crystallinity of PEEK, and thus its physicochemical properties, can be controlled through the MCM thermal drawing process. An important challenge that remains is to produce fully amorphous PEEK fibers that could potentially propagate light with reduced optical diffusion. In the present case, a threshold is reached and crystallinity values do not decrease below 9% despite the increase of cooling rate. Still, because the crystallinity of the thin PEEK fiber is low enough, it remarkably appears transparent as depicted on Fig. 5b,c. Propagation of light is tested through a few-centimeter-long portion of the bare PEEK core (retrieved from an MCM-I fiber after the glass cladding is removed). A 632 nm HeNe laser is used as the incident light source. Optical losses are estimated from the measurement of the intensity of the light longitudinally scattered from the PEEK fiber which is plotted on Fig. 5e. The data are extracted from the image shown on the inset of Fig. 5e using ImageJ software (version 1.53e, imagej.nih.gov). The attenuation is estimated to be around 3.2 dB/cm by fitting the data points with the Beer-Lambert law. This value is in the range of the losses measured on polymer-based specialty optical fibers and waveguides^38^. Owing to their good biocompatibility, PEEK fibers in short segments could be considered for applications in biophotonics as for instance for light diffusers or optical neural interfacing and others^39^.

## Conclusions

We have demonstrated in the present manuscript that the molten core thermal drawing technique applies to the production of fibered glass structures integrating continuous polymer inclusions. In particular, we have proven that unprecedented glass-polymer associations are enabled by the proposed methodology, which specifically opens the use of soft oxide glasses with amorphous or even semi-crystalline thermoplastics for fiber drawing. Several glass/polymer combinations in the fiber form are proposed with a focus on the association of poly ether ether ketone with tellurite and phosphate glasses. It is established that the degree of crystallinity of PEEK can be controlled through this original thermal drawing procedure, which demonstrates that the MCM not only opens new prospects for material shaping from the milli- to the nanometer scale, but also represents an innovative route for the tailoring of the physicochemical properties of polymers.

Future works will be devoted to the assembly of new functional glass fiber geometries which integrate mechanical, electrical, optical and so on, features through the use of polymers or polymer composites. In the highly dynamic context of multimaterial fiber development, we believe our findings could initiate further investigations to produce advanced hybrid, multi-purpose optical fibers.

## Data Availability

The datasets used and/or analyzed during the current study are available from the corresponding author upon reasonable request.
